# Editorial: 50 years of BMT: long-term outcomes and late complications after transplantation

**DOI:** 10.3389/fonc.2023.1253971

**Published:** 2023-08-01

**Authors:** Eleni Gavriilaki

**Affiliations:** 2^nd^ Propedeutic Department of Internal Medicine, Aristotle University of Thessaloniki, Thessaloniki, Greece

**Keywords:** stem cell transplant, allogeneic, hematopoietic, chronic graft-versus-host disease, 7 GvHD, secondary malignancies

## Editorial

Stem cell transplantation is a key and sometimes curative treatment approach for many hematological malignancies. Over the past couple of decades, the use of stem cell transplantation has expanded geographically and continues to evolve, with an increasing number of transplantations from unrelated donors, and novel regimens becoming available for prevention and management of transplant complications.

2022 marks the 50th anniversary of the Center for International Blood and Marrow Transplantation Registry (CIBMTR). Launched in 1972, doctors in the United States, the Netherlands, and France combined their efforts to create an international registry of transplants to monitor the outcomes of patients undergoing transplantation, with the goal of deducing the most effective approaches. In order to celebrate the significant advances which have been made in optimizing stem cell transplantation for patients with hematological malignancies over the past 50 years, Frontiers in Oncology is delighted to present three Research Topics, which together aim to collate the next phase of advances in these fields as Open Access publications.

This Research Topic is focusing on advances in understanding the long-term outcomes of patients who undergo bone marrow transplantation, in particular in relation to late complications. Late complications are fairly frequent in patients who undergo hematopoietic stem cell transplantation (HSCT), with long-term impacts on life expectancy. Secondary solid cancers and chronic graft-versus-host disease (GvHD), that which onsets 100 days after transplantation, are two of the most concerning late complications. Chronic GvHD is similar to autoimmune diseases, resulting in a gradual deterioration of quality of life and an increased risk of mortality (1).

For patients who survive for five years after HSCT, secondary cancers are the primary cause of death, with the risk increasing over time. Non-squamous cell cancers have been associated with radiation exposure, and squamous cell cancers of the skin and oropharyngeal region have been associated with chronic GvHD. Furthermore, chronic GvHD is typically treated with immunosuppressants, which are known to promote carcinogenesis (2).

This Research Topic welcomed submissions contributing to improving and understanding the long-term outcomes for patients with hematologic malignancies undergoing bone marrow transplantation. All articles submitted to us for this Research Topic underwent a rigorous peer review process. Ultimately, four articles were published.

(i) Effect of autologous hematopoietic stem cell transplantation for patients with peripheral T-cell lymphoma in China was assessed using a propensity score-matched analysis (Gao et al.). This analysis of 414 patients suggested ASCT may improve long-term survival in patients transplanted at first complete or partial remission, in particular for those suffering from angioimmunoblastic T-cell lymphoma.

(ii) In refractory/relapsed acute B lymphocytic leukemia, long-term follow-up results of CAR-T therapy followed by allogeneic hematopoietic stem cell transplantation were analyzed (Li et al.). Interestingly, poor prognosis for progression-free and overall survival was predicted by germline EP300 and tumor somatic TP53 mutations.

(iii) Post-transplant cyclophosphamide (PTCy) *versus* anti-thymocyte globulin (ATG) in allogeneic hematopoietic stem cell transplantation from unrelated donors were compared in a systematic review and meta-analysis (Tang et al.). The six selected studies suggested that PTCy is associated with reduced incidence of grade II-IV or III-IV acute GvHD, non-relapse mortality and EBV-related complication, as well as better overall survival compared to ATG.

(iv) Long-term health outcomes of allogeneic hematopoietic stem cell transplantation were reviewed (Kelkar et al.), including cardiovascular, noninfectious pulmonary, gastrointestinal, renal, dermatologic, neurologic, psychiatric, ophthalmologic, and other complications. Their recognition and management constitute an important part of survivorship care.

Taking into account the multi-disciplinary character of this Research Topic, we hope that it will inspire researchers to continue their explorations into novel advances in their fields.

## Author contributions

EG: Writing – original draft, Writing – review & editing.
